# Identification of exosomal microRNA panel as diagnostic and prognostic biomarker for small cell lung cancer

**DOI:** 10.1186/s40364-023-00517-1

**Published:** 2023-09-13

**Authors:** Dong Ha Kim, Hyojeong Park, Yun Jung Choi, Kyungtaek Im, Chae Won Lee, Da-Som Kim, Chan-Gi Pack, Hyun-Yi Kim, Chang-Min Choi, Jae Cheol Lee, Wonjun Ji, Jin Kyung Rho

**Affiliations:** 1 Asan Institute for Life Sciences, 05505 Seoul, South Korea; 2 Department of Biomedical Sciences, AMIST, 05505 Seoul, South Korea; 3 Department of Convergence Medicine, University of Ulsan College of Medicine, 88, Olympic-ro 43-gil, Songpa-gu, Seoul, 05505 South Korea; 4 Department of Pulmonary Critical and Care Medicine, University of Ulsan College of Medicine, 88, Olympic-ro 43-gil, Songpa-gu, Seoul, 05505 South Korea; 5 Department of Oncology, Asan Medical Center, University of Ulsan College of Medicine, 05505 Seoul, South Korea; 6 NGeneS Inc, Asan-Si, Gyeonggi-do South Korea

**Keywords:** Exosome, Exosomal miRNA, Diagnosis, Prognosis, SCLC

## Abstract

**Background:**

Small cell lung cancer (SCLC) has an exceptionally poor prognosis; as most of the cases are initially diagnosed as extensive disease with hematogenous metastasis. Therefore, the early diagnosis of SCLC is very important and may improve its prognosis.

**Methods:**

To investigate the feasibility of early diagnosis of SCLC, we examined exosomal microRNAs (miRNAs) present in serum obtained from patients with SCLC. First, exosomes were isolated in serum from patients with SCLC and healthy individuals and were characterized using particle size and protein markers. Additionally, miRNA array was performed to define SCLC-specific exosomal miRNAs. Second, the obtained miRNAs were further validated employing a large cohort. Finally, the ability to diagnose SCLC was estimated by area under the curve (AUC), and intracellular mRNA change patterns were verified through validated miRNAs.

**Results:**

From the miRNA array results, we selected 51-miRNAs based on p-values and top 10 differentially expressed genes, and 25-miRNAs were validated using quantitative reverse transcription-polymerase chain reaction. The 25-miRNAs were further validated employing a large cohort. Among them, 7-miRNAs showed significant differences. Furthermore, 6-miRNAs (miR-3565, miR-3124-5p, miR-200b-3p, miR-6515, miR-3126-3p and miR-9-5p) were up-regulated and 1-miRNA (miR-92b-5p) was down-regulated. The AUC value of each miRNA sets between 0.64 and 0.76, however the combined application of 3-miRNAs (miR-200b-3p, miR-3124-5p and miR-92b-5p) remarkably improved the diagnostic value (AUC = 0.93). Gene ontology analysis revealed that the 3-miRNA panel is linked to various oncogene pathways and nervous system development. When the 3-miRNAs were introduced to cells, the resulting changes in total mRNA expression strongly indicated the presence of lung diseases, including lung cancer. In addition, the 3-miRNA panel was significantly associated with a poorer prognosis, although individual miRNAs have not been validated as prognostic markers.

**Conclusion:**

Our study identified SCLC-specific exosomal miRNAs, and the 3-miRNAs panel (miR-200b-3p, miR-3124-5p and miR-92b-5p) may serve as a diagnostic and prognostic marker for SCLC.

**Graphical abstract:**

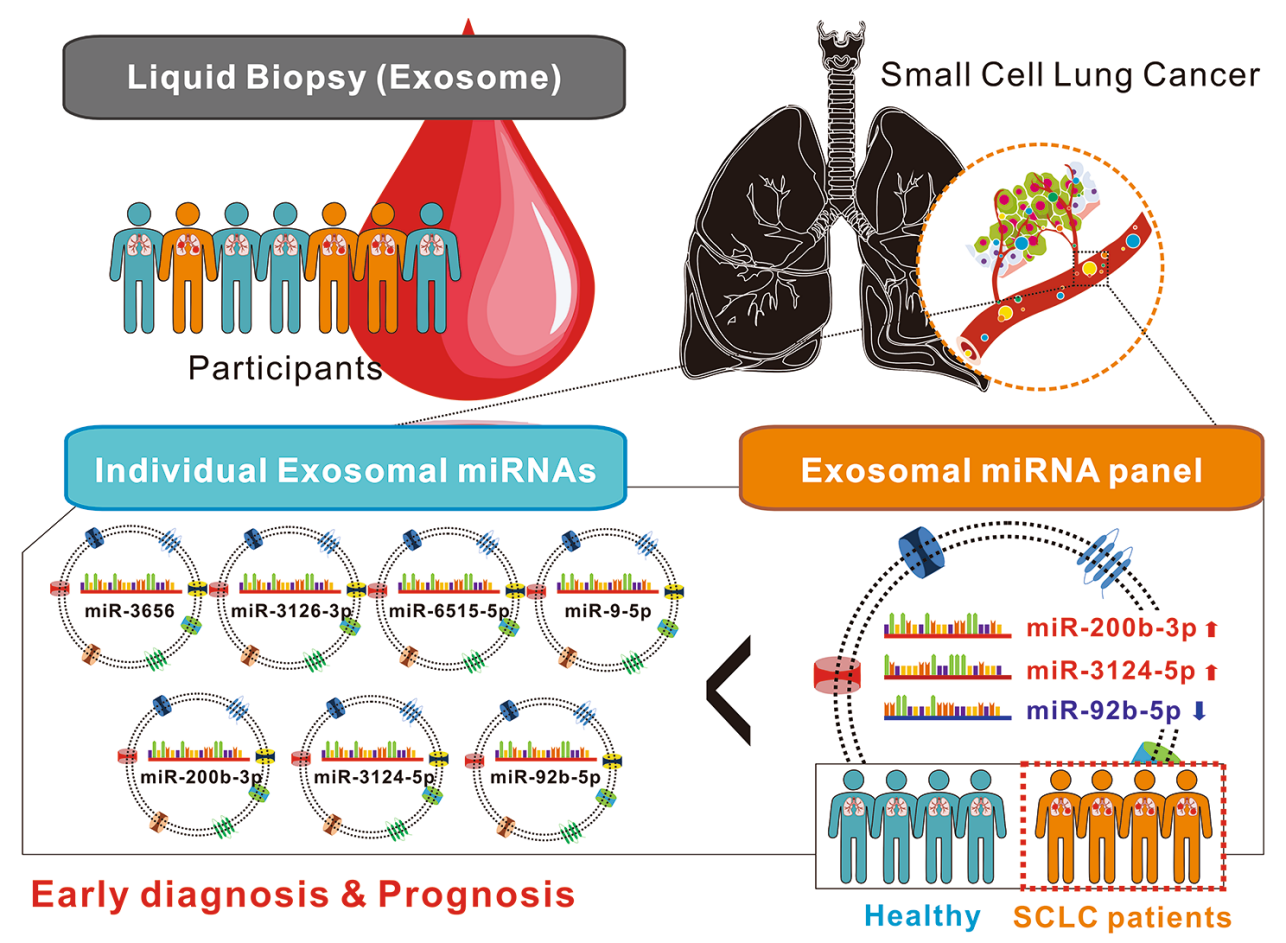

**Supplementary Information:**

The online version contains supplementary material available at 10.1186/s40364-023-00517-1.

## Background

The diagnosis of lung cancer is broadly classified into two main categories as thus: for small cell lung cancer (SCLC) and non-small cell lung cancer (NSCLC). Specifically, SCLC comprises approximately 15% of all lung cancer, and the general 5-year survival rate is < 10% [1, 2]. In contrast to NSCLC, for which the survival rate has dramatically improved in the past few decades because of targeted therapy and immunotherapy, major developments over standard platinum-based therapy have not yet been made in SCLC treatment [3]. In addition to the lack of treatment efficiency, most (approximately 70%) patients with SCLC are diagnosed as extensive disease (ED). Thus, the early detection of SCLC is quite necessary to improve the clinical outcome of patients with SCLC.

Exosomes are 30–150 nm endocytic membrane-derived vesicles that play important roles in cell-to-cell communication [4–6]. They act as a transporting system for various molecular constituents of their cellular origin, including proteins, lipids, DNA, mRNA, and miRNA [4–6]. Although exosome is initially considered as “garbage bags” for cells to get rid of unwanted constituents, presently it is known to participate in many physiological and pathological processes, such as immune regulation, cell differentiation, epigenetic regulation, and tumor occurrence and progression [7–10]. Importantly, accumulating data have revealed that exosomes are associated with various stages of tumor progression, including immune regulation, angiogenesis, metastasis, drug resistance, and cell proliferation [11–20]. Therefore, any enriched component within exosomes may play specific role in tumor biology.

In addition to the biological roles of exosome, they can aid in drug delivery and diagnosis. In diagnostic field, many papers have proposed the application of exosomes as diagnostic tools; as they contain biomarkers for disease [21–26]. Furthermore, these applications of exosome have great advantages, including its feasibility for all biofluids, high biological stability, and long monitoring of chronic disease. Although exosome-based strategies for disease diagnosis are yet difficult owing to in-depth validation of biomarker and isolation and characterization of exosome, these trials could amount to success through fast development of technology.

In this study, we isolated exosomes in serum obtained from patients with SCLC and healthy individuals, and analyzed miRNAs to determine the SCLC-specific exosomal miRNAs. Furthermore, we explored whether the combination of multiple miRNAs could predict SCLC diagnosis.

## Materials and methods

### Clinical specimens

A total of 126 serum samples were collected from 28 limited disease (LD)-SCLC, 48 extensive disease (ED)-SCLC and 50 healthy individuals after an informed consent was obtained in each case. LD-SCLC and ED-SCLC were defined according to the NCCN guidelines for SCLC [27]. The baseline characteristics of patients with SCLC are shown in Table 1. These analyses were approved by the Asan Medical Center Institutional Review Board (2016 − 0752, 2018 − 0462), which exempted the requirement for informed consent due to their retrospective nature.

**Table 1:** Clinicopathologic characteristics of patients

Variable	Analytic set			Test set	
	Control	SCLC		Normal	SCLC
**Patients, n**	5	5		50	76
**Age**	61.8 ± 5.3	59.2 ± 7.2		55.88 ± 9.7	64.2 ± 8.2
**Male sex (%)**	5 (100.0)	5 (100.0)		27 (54.0)	67 (88.2)
**Smoking status**					
Never, n (%)	·	·		28 (56.0)	5 (6.6)
Former/current, n(%)	5 (100.0)	5 (100.0)		22 (44.0)	71 (93.4)
Smoking amound, pack/year, mean ± SD	30.14 ± 10.3	58 ± 27.7		14.16 ± 19.2	33.79 ± 19.0
**Underlying comorbidities**	2	4		29	50
Respiratory diseases, n(%)	·	2 (40.0)		2 (4.0)	20 (26.3)
Cardiovascular diseases, n(%)	2 (40.0)	4 (80.0)		22 (44.0)	35 (46.0)
Other medical conditions, n(%)	1 (20.0)	2 (40.0)		17 (34.0)	17 (22.4)
**STAGE**					
Limited, n (%)	·	5 (100.0)		·	28 (36.8)
Median OS (95% CI), months	· 30 (14.0 to 36.4)			· 25 (23.2 to 34.4)	
Extensive, n (%)	·	·		·	48 (63.2)
Median OS (95% CI), months	·	·		· 10 (9.9 to 15.6)	
**Tumor invasion**					
T1 + T2	·	2 (40.0)		·	32 (42.1)
T3 + T4	·	3 (60.0)		·	44 (57.9)
**Lymph node metastasis**					
Negative	·	·		·	11 (14.5)
Positive	·	5 (100.0)		·	65 (85.5)
**Distant metastasis**					
Negative	·	5 (100.0)		·	32 (42.1)
Positive	·	·		·	44 (57.9)
**Treatment modality**					
CCRT	·	4 (80.0)		·	30 (39.5)
Chemotherapy	·	1 (20.0)		·	46 (60.5)
**Surgery**					
Yes	·	·		·	·
No	·	5 (100.0)		·	76 (100.0)

Abbreviations: SCLC, small cell lung cancer; OS, overall survival; CCRT, concurrent chemoradiotherapy Data represent mean (± SD) unless otherwise stated

### Exosome isolation

Isolation of exosomes from serum samples were performed as previously described method [16]. Briefly, thawed serum samples were centrifuged at 10,000 × *g* for 30 min at 4 °C to completely eliminate cellular debris. Thereafter, the supernatant was centrifuged at 100,000 × *g* for 70 min at 4 °C. The pellets were washed with phosphate-buffered saline (PBS), ultracentrifuged, and re-suspended in PBS. Subsequently, isolated exosomes were quantified using a standard protein assay (Bio-Rad Laboratories, Hercules, CA) and stored at − 80 °C until needed.

### Negative staining electron microscopy

The extracted exosomes were fixed in 2% (vol/vol) paraformaldehyde for 5 min at room temperature. After fixation, 10 µg of exosome suspension was applied to formvar/carbon-coated grids (200 mesh) for 3 min and stained with 2% uranyl acetate. Following the removal of excess uranyl acetate with filter paper, the grid was observed with a transmission electron microscope (TEM, Hitachi H7600, Japan) at 80 kV.

### Nanoparticle tracking analysis

Particle concentration and size of serum-derived exosomes were analyzed using NanoSight NS300 system (Malvern Instruments Ltd, Malvern, UK), which enables the tracking of Brownian motion of nanoparticles in a liquid suspension on a particle-by-particle basis. Briefly, the samples were diluted 100–500-fold in PBS to reduce the number of visible particles to less than 100 per frame, and readings were taken 3 times over a 60 s at 10 frames per second at room temperature. Thereafter, data were analyzed using a nanoparticle tracking analysis software (NTA version 2.3 build 0017).

### Western blot analysis

Total proteins from exosomes were prepared in EBC lysis buffer (50-mM Tris–HCl [pH 8.0], 120-mM NaCl, 1% Triton X-100, 1-mM EDTA, 1-mM EGTA, 0.3-mM phenylmethylsulfonylfluoride, 0.2-mM sodium orthovanadate, 0.5% NP-40, and 5-U/mL aprotinin) and quantified using the Bradford method. Approximately 20 mg of protein was separated by SDS-PAGE and transferred to PVDF membrane (Invitrogen, Carlsbad, CA) for western blot analysis. Membranes were probed using antibodies against TSG101 (ab125011, 1:1000, Abcam, Cambridge, UK), HSP90 (BD610418, 1:2000, BD Biosciences), CD63 (sc-5275, 1:1000, Santa Cruz Biotechnology, Santa Cruz, CA), CD81 (sc-7637, 1:1000, Santa Cruz Biotechnology) as the primary antibody. Subsequently, the membranes were treated with a horseradish peroxidase-conjugated secondary antibody. All signals were developed using an enhanced chemiluminescence system (Thermo Scientific, Rockford, IL).

### MicroRNA microarray

Serum-derived exosomal RNA containing miRNAs from 5 patients with SCLC and 5 controls with lung nodules was extracted using the miRNeasy mini kit (Qiagen, Hilden, Germany) in accordance with the manufacturer’s instructions. The quality of RNA was assessed using Agilent 2100 bioanalyzer with the RNA 6000 Pico Chip (Agilent Technologies, Amstelveen, The Netherlands), and RNA quantification was performed applying a NanoDrop 2000 spectrophotometer system (Thermo Fisher Scientific, Waltham, MA, USA). The exosome miRNA expression profiles were established using the Affymetrix GeneChip® miRNA 4.0 Array (Affymetrix, Santa Clara, CA) based on miRBase v.20 (http://www.mirbase.org). Subsequently, miRNA microarray was prepared, hybridized, and scanned by a local authorized Illumina array service provider (Macrogen, Seoul, South Korea). All procedures were performed in accordance with the manufacturer’s recommendations. To analyze the differentially expressed miRNAs, quantile normalization was performed to standardize the data throughout the samples.

### Quantitative real time reverse transcription-polymerase chain reaction (qRT-PCR)

The qRT-PCR for the identification of serum-derived exosomal miRNAs was performed as previously described [18]. Total RNA extracted for quantification of mature miRNAs was polyadenylated with a poly (A) tailing kit (Ambion, Austin, TX) and poly (T) adaptor prior to reverse transcription. Quantitative real-time RT-PCR analysis was performed applying ABI 7900 real-time PCR System using the SYBR Green Master Mix (Applied Biosystems, Foster City, CA) with the miRNAs primer set according to the manufacturer’s protocol. Notably, let-7a-5p was used as a loading control. The primers used are listed in Supplementary Table 1.

### Bioinformatics analysis

Overlay analysis was performed using Vesiclepedia (http://microvesicles.org/) and ExoCarta (http://exocarta.org/) exosomal miRNA databases to compare the miRNA array data sets obtained [28, 29]. Briefly, these databases provide an overview of miRNAs identified in EVs, including exosomes of various sample types. TargetScan8.0 (http://www.targetscan.org/), miRDB (http://www.mirdb.org/) and miRWalk target prediction (http://www.mirwalk.umm.uni-heidelberg.de/) algorithms were used to predict the target genes of these prognostic exosomal miRNAs. The miRNA‑target gene interaction networks were visualized using Cytoscape v3.4.0. Functional enrichment of these target genes was performed using the Kyoto Gene and Genome Encyclopedia (KEGG), Gene Ontology (GO) and Disease Ontology (DO) databases.

### Cell culture and transfection

The human bronchial epithelial cell line BEAS2B were cultured in RPMI1640 medium, supplemented with 10% fetal bovine serum (FBS), 100-U/mL penicillin and 100-mg/mL streptomycin (Invitrogen, Carlsbad, CA, USA) at 37 °C, 95% humidified air, and 5% CO2. The miR-3124-5p mimic (MC17992), miR-200b-3p mimic (MC10492), miR-92b inhibitor (MH12985), scrambled miRNA controls were chemically synthesized by Applied Biosystems (Applied Biosystems, Foster City, CA, USA). When BEAS2B cells reached 60% confluence, each miRNA mimic and inhibitor or scrambled miRNA controls (final concentration of 50 nM) were transfected using Lipofectamine 2000 according to the manufacturer’s instructions (Invitrogen). Cells were incubated at 37 °C in a CO2 incubator for 48 h before RNA extraction.

### Prediction models

Tidymodels (v0.1.4) [30], a collection of R packages for modeling and machine learning, were employed to predict the k-nearest neighbors models. The expression of 7 miRNAs (predictors) and diagnosis result (outcome) of 126 biopsy samples (normal = 50, SCLC = 76) were used to develop the model. The samples were randomly selected and separated into training (94) and validation (32) set. All possible combinations of predictors with number of neighbors 3, 5, 7 were examined. The developed model was validated by diagnostic ability assessed via receiver operating characteristic (ROC) and area under the curve (AUC) calculation. Statistical significance of AUC was accessed using a method suggested by Mason and Graham [31]. To predict risk score, a Cox proportional hazards regression model was developed using a package for survival analysis in R; survival (v 3.3-1) [32]. After fitting the model with expression of 3 miRNAs (miR-3124-5p, miR-92b-5p, and miR-200b-3p) in biopsy and survival information of 76 patients with SCLC, the risk score of each patient was calculated. The median of the risk scores (505.3) was used as a criterion to determine whether the patient belongs to low- or high-risk group. All analysis and visualization were performed under the R (v4.1.2) and R studio environment v2022.07.1 + 554 for Windows).

### Library preparation and mRNA-sequencing

Libraries were prepared from total RNA using the NEBNext Ultra II Directional RNA-Seq Kit (NEW ENGLAND BioLabs, Inc., UK). The isolation of mRNA was performed using the Poly(A) RNA Selection Kit (LEXOGEN, Inc., Austria). The isolated mRNAs were used for the cDNA synthesis and shearing, following manufacture’s instruction. Indexing was performed using the Illumina indexes 1–12. The enrichment step was carried out using of PCR. Subsequently, libraries were checked using the TapeStation HS D1000 Screen Tape (Agilent Technologies, Amstelveen, The Netherlands) to evaluate the mean fragment size. Quantification was performed using the library quantification kit using a StepOne Real-Time PCR System (Life Technologies, Inc., USA). High-throughput sequencing was performed as paired-end 100 sequencing using NovaSeq 6000 (Illumina, Inc., USA).

A quality control of raw sequencing data was performed using FastQC [33]. Adapter and low quality reads (< Q20) were removed using FASTX_Trimmer and BBMap [34, 35]. Then the trimmed reads were mapped to the reference genome using TopHat [36]. The RC (Read Count) data were processed based on FPKM + Geometric normalization method using EdgeR within R [37]. FPKM (Fragments Per kb per Million reads) values were estimated using Cufflinks [38].

### Statistical analysis

Categorical variables were analyzed using the Pearson’s chi-square test or Fisher’s exact test. Continuous variables were analyzed using a student’s t-test or analysis of variance. Overall survival curves were plotted using the Kaplan–Meier method and were compared using a log-rank test. All tests of significance were two-sided, and the differences between groups were considered to be significant when the *p*-value was < 0.05. All statistical analyses were performed using GraphPad Prism version 8.0 (GraphPad Software Inc. CA, USA).

## Results

### Characteristic similarity of non-cancerous nodules and exosomes in patients with SCLC

**Fig. 1:**
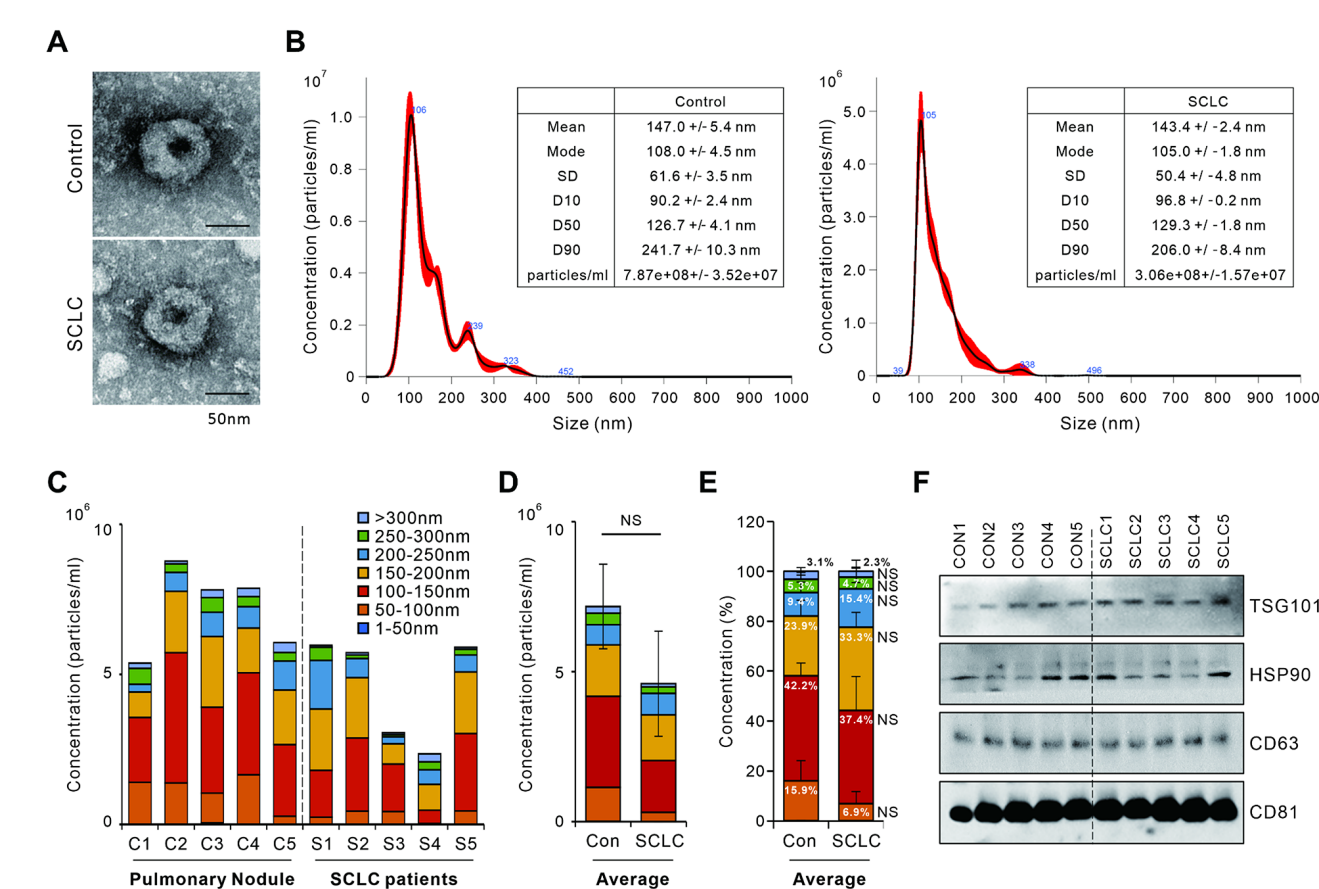
Characterization of serum-derived exosomes from patients with small cell lung cancer (SCLC) and non-cancerous lung nodule. Serum-derived exosomes were isolated via ultracentrifugation as described in the methods. (**A**) Representative transmission electron microscopy images of serum-derived exosomes. (**B**–**D**) Size distribution and concentration of the isolated particles determined by nanoparticle tracking analysis (NTA). (**E**) Stacked bar graph showing percentage by exosome size. (**F**) Western blot analysis of serum-derived exosomal proteins to verify the expression of exosome biomarkers. Data are mean ± SDs; n = 5 per group

To investigate the feasibility of diagnosis and prognosis through SCLC-specific exosomal miRNA, clinical specimen recruitment was conducted. The sample collection was designed as an “Analytic set” for exosomal miRNA array analysis and a “Test set” for validation. The “Analytic set” consisted of serum from 5 non-cancerous lung nodules (control) and 5 samples from patients with SCLC for exosomal miRNA array analysis. The “Test set” consisted of 50 healthy individuals and 76 SCLC serum (28 LD-SCLC, 48 ED-SCLC) used to validate analyzed miRNA expression and establish a diagnostic model (Table 1). Exosomes exhibited the characteristics of typical exosomes with a distinctive round cup-shaped morphology through negative staining TEM images (Fig. 1A) [39]. According to NanoSight analysis, the mode size of the collected particles was observed to be 100–120 nm in diameter (Fig. 1B). Most of the particles exhibiting a heterogeneous size range between 50 and 500 nm in diameter were within the normal range of the exosome size (30–200 nm in diameter) (Fig. 1C) [40, 41]. Additionally, there were no significant differences in the quantitative and ratiometric comparisons by exosome size between the control and SCLC groups (Fig. 1D-E). In addition, the expression of exosomal markers TSG101, HSP90, CD63 and CD81 in individual patient specimens were confirmed by western blot (Fig. 1E). Overall, these results show that there was no significant difference in morphological, size variation, and amount between the control and SCLC exosome.

### Significant differences in exosomal miRNA profiling between non-cancerous nodules and patients with SCLC

**Fig. 2:**
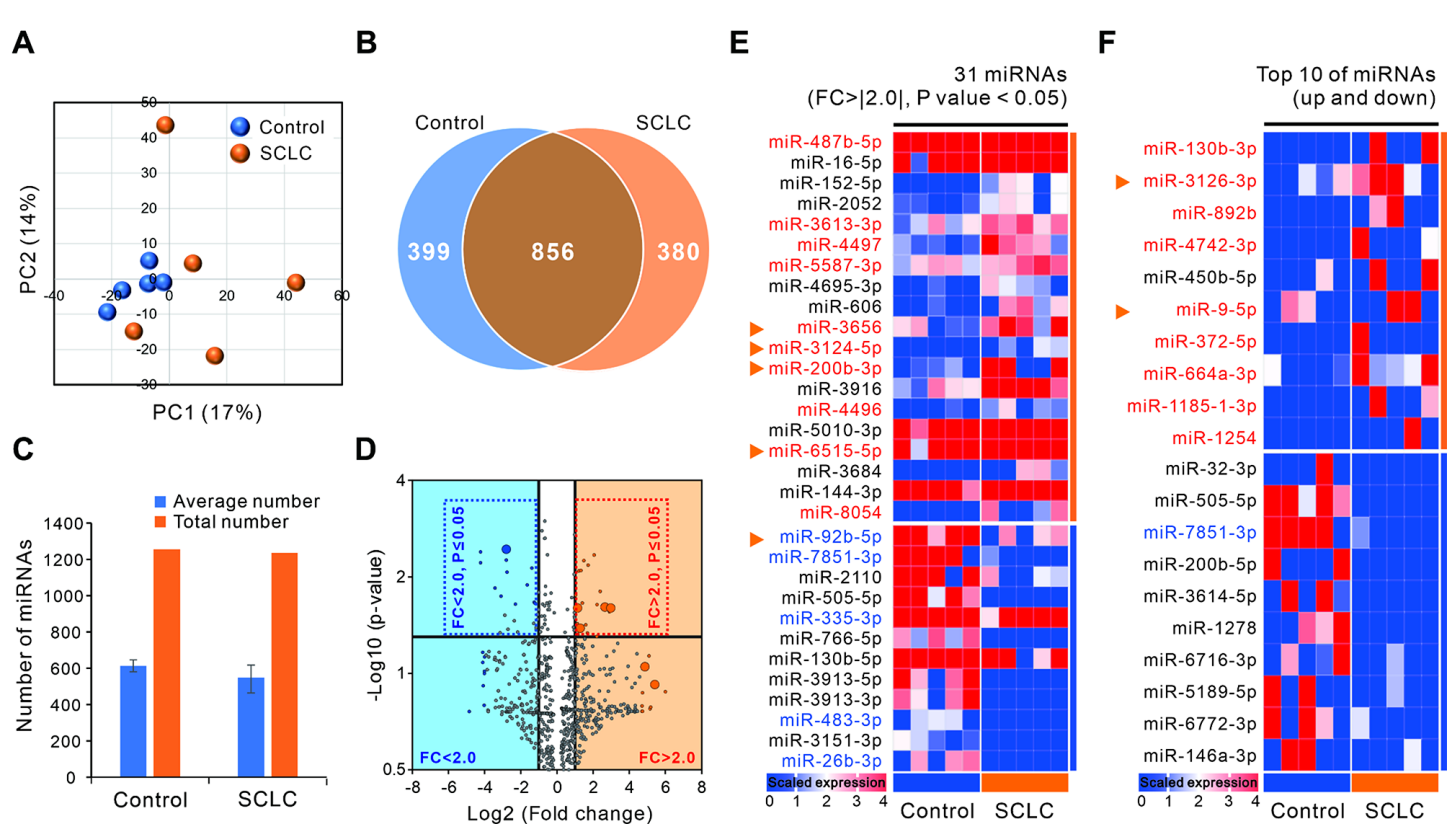
Identification of exosomal microRNA (miRNA) expression profiles in patients with SCLC. The miRNA array was performed using serum-derived exosomes from non-cancerous lung nodules (control) and patients with SCLC (n = 5 per group). (**A**) Principal component analysis (PCA) plot of merged miRNA array data sets. (**B**) Venn diagrams show the number of all miRNAs differentially expressed between groups. (**C**) The average or total number of miRNAs identified through the miRNA array. (**D**) Volcano plots showing the distribution of 2-fold up- and down-regulated miRNAs. The seven exosomal miRNAs validated in the large cohort are shown as colored dots (blue: FC < 2.0, orange: FC > 2.0). (**E**) Heatmap analysis showing 31 exosomal miRNAs reveals significantly more than 2-fold up- and down-regulation (*p* < 0.05). (**F**) Ten exosomal miRNAs with the highest or lowest expression, respectively in patients with SCLC and non-cancerous lung nodules (controls). The 25 miRNAs validated in the “Analytic set” are shown in red and blue (red: upregulated, blue: downregulated), and the seven miRNAs validated in the “Test set” are indicated by orange arrows

Total RNA isolated from exosomes was analyzed using an Agilent bioanalyzer small RNA chip. Most of the exosomal RNA sizes were less than 200 nt (Figure S1). This indicates that exosomes are rich in short RNAs, including miRNAs. Through miRNA arrays, we screened exosomal miRNAs that were distinct between non-cancerous lung nodules (control) and of patients with SCLC. In the principal component analysis of standardized gene expression data, the clustered control group exhibited low sample-to-sample variability, as expected of disease-free specimens. In contrast, the SCLC group showed sporadic distribution, indicating the degree of genetic diversity, compared to the control group (Fig. 2A).

Through the miRNA array results, we identified 1255 miRNAs in the control, 1236 miRNAs in SCLC, and 856 miRNAs commonly expressed among them (Fig. 2B). Altogether, approximately 1200 miRNAs were detected in the exosomes of each group and an average of approximately 600 miRNAs were expressed in each patient, with no significant difference in the number of miRNAs between the two groups (Fig. 2C). We identified several cross-referenced miRNAs in publicly available Exosome databases (Vesiclepedia and ExoCarta). As a result, over 90% of miRNAs in our microarray data were previously presented with extracellular functions, and we identified 135 novel exosomal miRNAs that were not reported to be present in exosomes (Figure S2). Volcanic plots identified various miRNAs differentially expressed in serum-derived exosomes of SCLC compared to control (Fig. 2D). The heatmap revealed 31 exosomal miRNAs with at least 2-fold significant (FC > |2.0| and *p*-value < 0.05) expression differences, with 19 miRNAs up-regulated and 12 miRNAs down-regulated in the SCLC compared to control (Fig. 2E). In addition, each of the 10 miRNAs (total of 20 miRNAs) with the greatest increase or decrease according to the expression rate regardless of the *p* value were plotted (Fig. 2F). Through this process, we selected serum-derived exosomal miRNAs specific for SCLC patients that are distinct from the control.

### Validation of exosomal miRNA profiles in a large cohort

**Fig. 3:**
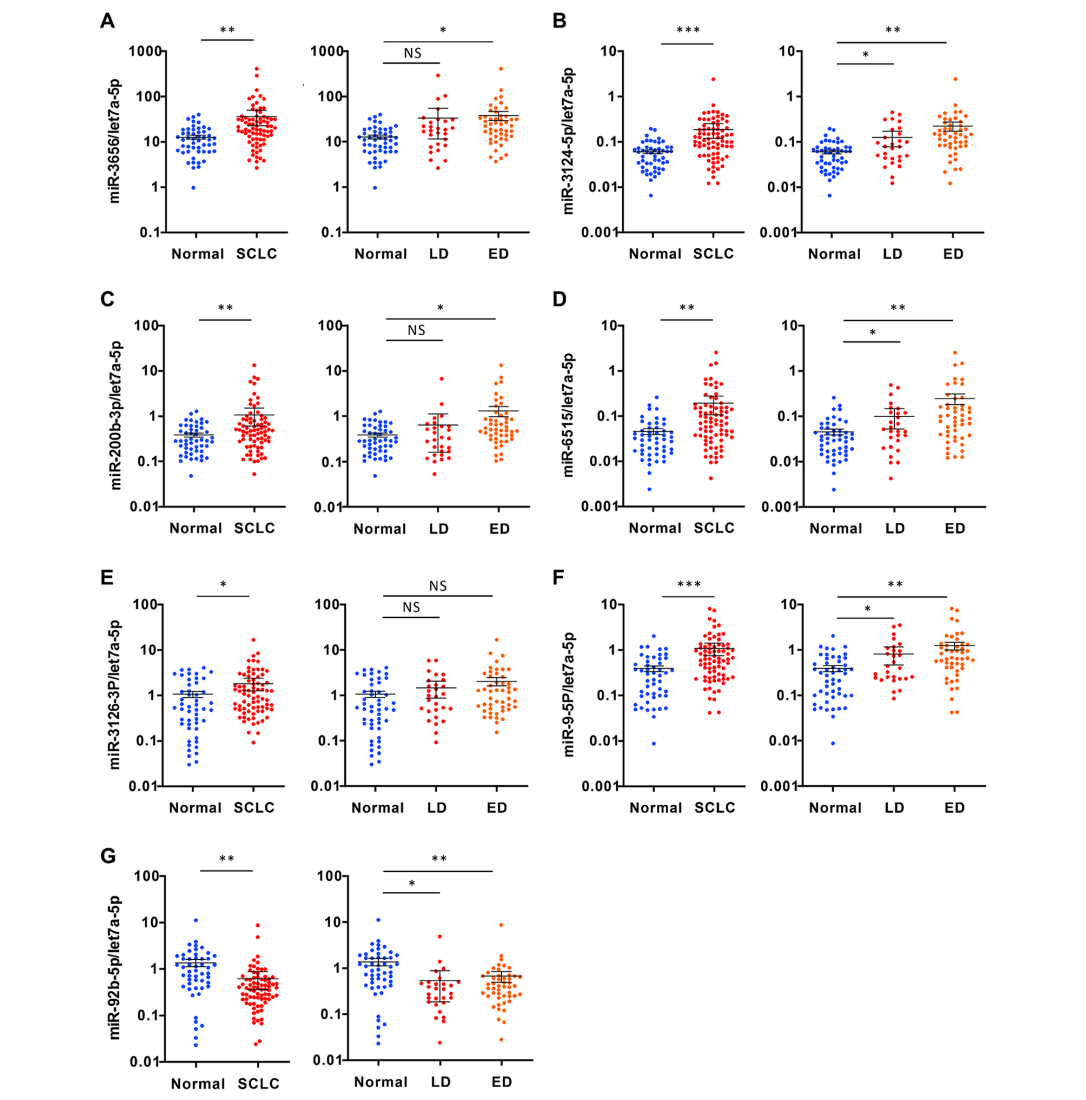
Validation of differentially expressed exosomal miRNAs. Quantitative real-time PCR was performed using serum-derived exosomes from 28 LD-small cell lung cancer (SCLC), 48 ED-SCLC, and 50 healthy participants. (**A**) Expression levels of has-miR-3656; (**B**) has-miR-2124-5p; (**C**) has-miR-200b-3p; (**D**) has-miR-6515; (**E**) has-miR-3126-3p; (**F**) has-miR-9-5p, and (**G**) has-miR92b-5p. The data were analyzed using Brown–Forsythe and Welch ANOVA with Dunnett’s T3 multiple comparison, comparing each experimental condition with the control. Let-7a-5p was used as an internal control. Data are mean ± SDs; *p < 0.05; **p < 0.005; ***p < 0.0005

We obtained 51 candidate exosomal miRNAs from miRNA array analysis performed on the “Analytic set” and selected 25 miRNAs validated via qRT-PCR analysis using the same RNA samples (Figure S3). For validation of selected 25 miRNAs, we performed qRT-PCR in a larger cohort of “Test set” (50 healthy individuals and 76 SCLC patient serum derived exosomes). Prior to analysis, we confirmed the expression of let-7a-5p, RNU6B and RNU48 selected based on literature in a randomized cohort of 50 serum samples for selection of housekeeping miRNAs. As a result of the test, let-7a-5p showed the most equivalent concentration in the normal and SCLC exosome groups, which was consistent with the previous results (Figure S4) [17, 18]. As a result of qRT-PCR in the “Test set,“ we found 7 miRNAs with significantly different expression between the normal and SCLC among the 25 exosomal miRNAs. Six miRNAs (miR-3656, miR-3124-5p, miR-200b-3p, miR-6515, miR-3126-3p and miR-9-5p) were up-regulated, and one miRNA (miR-92b-5p) was down-regulated in SCLC exosome compared to the normal exosome. In addition, four miRNAs (miR-3124-5p, miR-6515, miR-9-5p and miR-92b-5p) showed significant differences in both patients with LD and ED, and the remaining 3-miRNAs (miR-3656, miR-200b-3p and miR-3126-3p) showed a significant difference only in patients with ED alone (Fig. 3A-G). All seven identified exosome miRNAs did not affect the survival rate of SCLC (Figure S5). Additionally, we analyzed exosomes from the sera of 50 healthy participants (28 non-smokers, 11 ex-smokers, and 11 current smokers) to confirm the association between the seven exosomal miRNAs and smoking. No significant differences in miRNA profiles were found between the groups (Figure S6). Through this validation process, we finally selected SCLC exosomal miRNAs that were distinct from normal.

### Construction of SCLC diagnostic model through exosomal miRNA combination

**Fig. 4:**
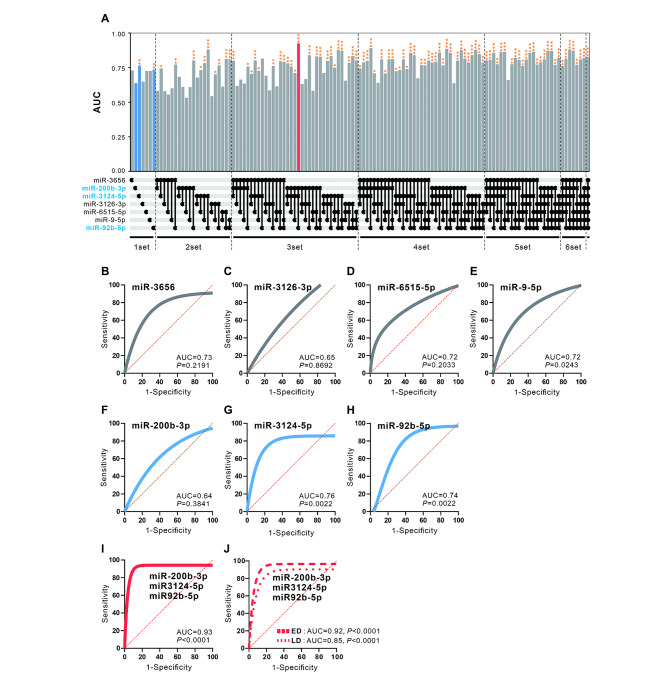
Evaluation of diagnostic efficacy by exosomal miRNA combination. Receiver operating characteristics (ROC) were performed on individual and combinatorial sets of 7 miRNAs validated in healthy individuals and patients with small cell lung cancer (SCLC). (**A**) Area under the ROC curve (AUC) in individual miRNAs and combination sets. ROC curves for classifying serum-derived exosomes were produced using each miRNA expression value for (**B**) miR-3656; (**C**) miR-200b-3p; (**D**) miR-3124-5p; (**E**) miR-3126-3p; (**F**) miR-6515-5p; (**G**) miR-9-5p, and (**H**) miR-92b-5p separately, and for (**I**) miR-200b-3p, miR-3124-5p and miR-92b-5p combined. The ROC curve for the miRNA sets were generated based on the predicted probability for each patient. *p < 0.05; **p < 0.005; ***p < 0.0005

The ROC curve is a diagram showing the true-positive rate (TPR, sensitivity) and the false-positive rate (FPR, 1-specificity) for multiple decision-making reference points based on the results of diagnostic tests [42, 43]. We performed ROC analysis to estimate the accuracy of the SCLC diagnostic test using preselected 7 exosomal miRNAs. Logistic regression was used to determine the best combination of miRNAs to diagnose SCLC. Binary logistic regression of all combinations through 7 miRNAs was used, and ROC curves were constructed to determine the specificity and sensitivity of miRNA expression. Among all 92 combinations, the highest area under the ROC curve (AUC) values and p-values were obtained when using 3-miRNA linear combination models (miR-200b-3p, miR-3124-5p and miR-92b-5p) (Fig. 4A). As a result of evaluating 3 miRNA-combined panel (miR-200b-3p, miR-3124-5p and miR-92b-5p; AUC = 0.93, *p* < 0.0001) through ROC curve analysis, it was found that the performance of the diagnostic model was significantly improved compared to individual miR-200b-3p (AUC = 0.64, *p* = 0.3841), miR-3124-5p (AUC = 0.76, *p* = 0.0022) and miR-92b-5p (AUC = 0074, *p* = 0.0022) (Fig. 4B-I). In addition, ROC analysis was performed by dividing 76 SCLC samples into 28 LD-SCLC and 48 ED-SCLC to confirm the possibility of early diagnosis of SCLC through the three-miRNA combined panel. This panel showed a high AUC value in patients with LD and even higher values in patients with ED (AUC = 0.92, *p* < 0.001 for ED vs. AUC = 0.85, *p* < 0.001 for LD; Fig. 4J). We propose that the combination of 3-miRNAs (miR-200b-3p, miR-3124-5p and miR-92b-5p) validated by ROC analysis is the most ideal model for SCLC diagnosis.

### Prediction of SCLC prognosis through the combination of exosomal miRNAs

**Fig. 5:**
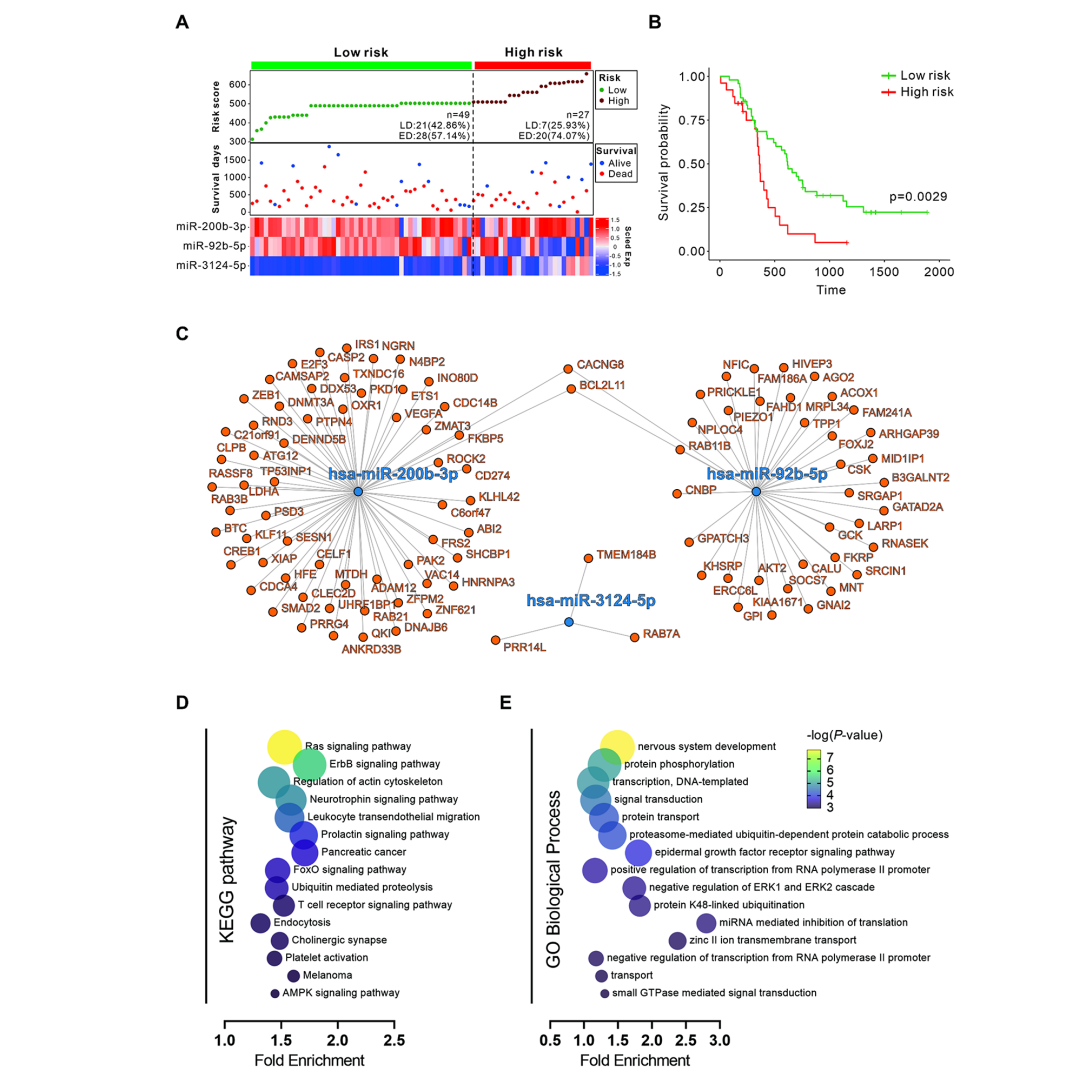
Construction of exosomal miRNA prognostic risk model in small cell lung cancer (SCLC) and functional evaluation of target genes. (**A**) Prognostic risk score model analysis with risk score, survival status distribution, and heat maps of 3 prognostic miRNAs in patients with low-risk and high-risk SCLC. (**B**) Kaplan–Meier survival curves of the low-risk and high-risk groups. (**C**) Interaction networks of the prognostic exosomal miRNAs and target genes. (**D**) Kyoto Encyclopedia of Genes and Genomes (KEGG) pathway and (**E**) Gene ontology enrichment analysis for target genes functional evaluation of exosomal miRNAs.

To present a comprehensive prognostic risk model, risk score, survival days, and miRNAs expression was constructed. Patients with SCLC were classified into the low-risk [49 patients; LD: 21 (42.86%), ED: 28 (57.14%)] and high-risk groups [27 patients; LD: 7 (25.93%), ED: 20 (74.07%)] according to the median risk score of previously acquired miRNAs (miR-200b-3p, miR-3124-5p, and miR-92b-5p) (Fig. 5A). Overall survival analysis showed that patients in the high-risk group were significantly associated with poor clinical outcomes and an increased risk of death compared to the low-risk group (Fig. 5B). These survival rate analysis contrasts with the previous results that there was no difference in survival rate according to individual miRNA expression and shows that the patient’s prognosis can be predicted through the combination of exosomal miRNAs (miR-200b-3p, miR-3124-5p, and miR-92b-5p). Additionally, miRNA target genes were derived using TargetScan8.0, miRDB and miRWalk online databases to identify functional parts of 3-miRNAs (miR-200b-3p, miR-3124-5p, miR-92b-5p). Overlapping genes from three online databases were considered as miRNA target genes, and the interaction network was visualized (Fig. 5C). Enrichment analysis of target genes was performed using DAVID v6.8. KEGG enrichment results suggested significant correlations with Ras, ErbB, neurotheophin and T cell receptor signaling pathways (Fig. 5D), and GO term enrichment analysis indicated that the target genes were significantly correlated with nervous system development, protein phosphorylation, and EGFR signaling pathways (Fig. 5E). These results suggest that this prognostic model through the combination of exosomal miRNAs can be used as a factor that can indicate the viability of SCLC.

### Integrated analysis of miRNAs and mRNA expression profiles in pathogenesis

**Fig. 6:**
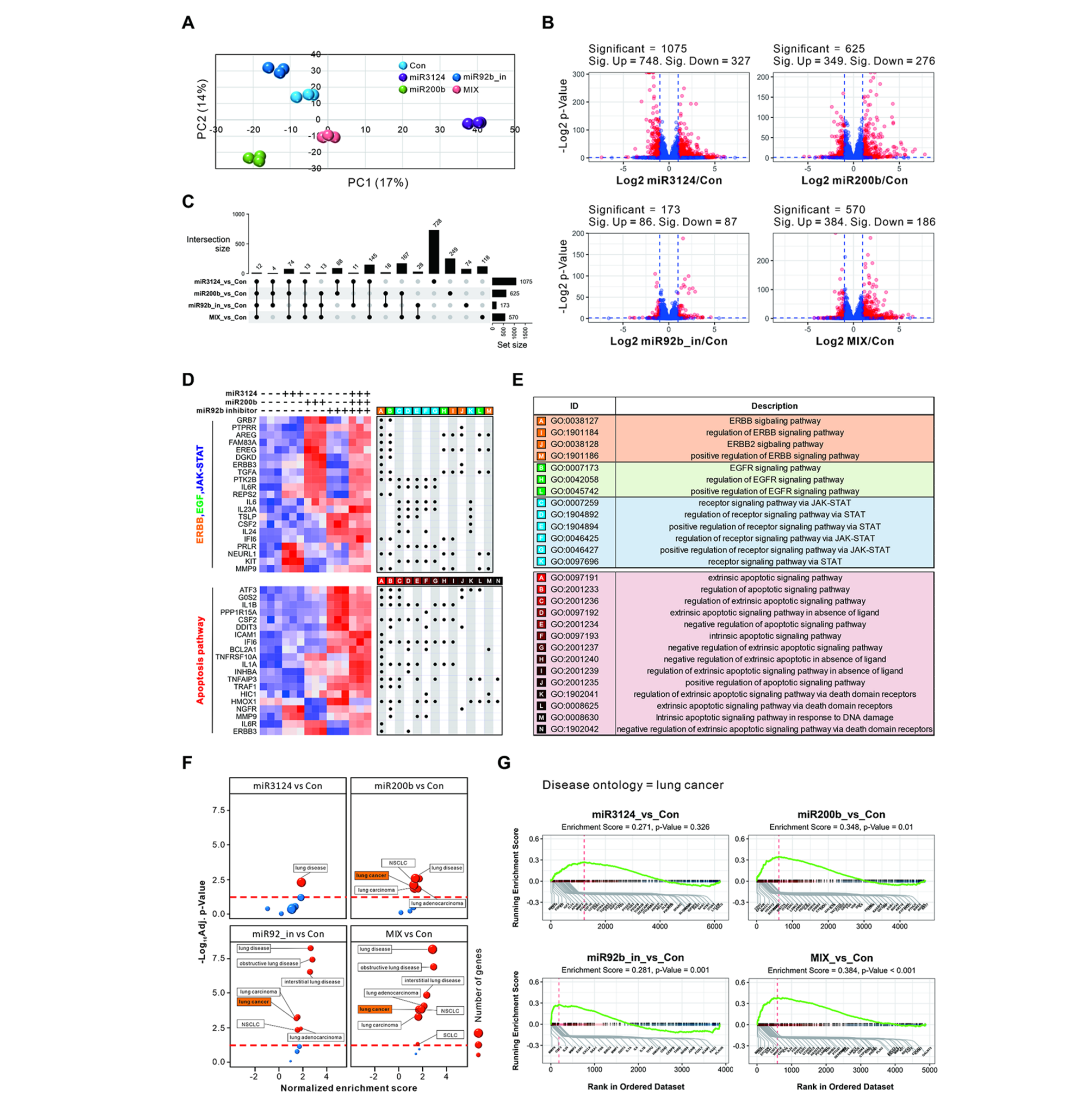
Functional characterization of integrated miRNAs in normal lung cell line. Merged mRNA-sequencing datasets were generated from a normal lung cell line (BEAS2B) transfected with individual miR3124_mimic, miR200b_mimic, and miR92b_inhibitor, or mixed sets of 3-miRNAs. (**A**) Principal component analysis (PCA) plot displaying the merged mRNA-sequencing data sets. (**B**) Volcano plots illustrating the distribution of significantly up- and down-regulated mRNAs (*p* < 0.05) by more than 2-fold. (**C**) Upset plots showing the number of all differentially expressed mRNAs between groups. (**D**) Heat map showing differentially expressed genes in ERBB, EGF, JAK-STAT, and Apoptosis pathways. The gene ontology (GO) term associated with each gene was indicated with a dot, and (**E**) the GO term for each pathway is described (ERBB [Orange: A,I,J,M], EGF [Green: B,H,L], JAK-STAT [Sky: C-G,K], and Apoptosis [Red: A-N]). (**F**) Bubble plot to visualize disease ontology (DO) terms related to lung disease. All bubbles represent gene sets. The larger the bubble, the greater the number of genes in the gene set. (**G**) DO plot based gene set enrichment analysis (GSEA) of lung cancer terms

To confirm the functional roles of the miRNAs identified through the SCLC diagnostic model, a normal lung cell line (BEAS2B) was transfected with each miRNA (miRcontrol, miR3124_mimic, miR200b_mimic, and miR92b_inhibitor) or the 3-miRNA mixed group, followed by mRNA-sequencing. Upon performing principal component analysis, no difference was found between each replicate. A significant difference was found in the mRNA profiles between the groups (Fig. 6A). Through differentially expressed gene analysis, we identified mRNAs from individual miRNA groups and the 3-miRNA mixed group. Each group was calibrated as a control, and a significance criterion (fold change cut-off of 2 and *p*-value cut-off of 0.05) was established and visualized as a volcano plot (Fig. 6B). The number of differentially expressed genes between the groups was cross-classified and visualized as an upset plot (Fig. 6C). The list of significant genes in each group was analyzed using Pathway-related Gene Ontology (GO) analysis (Figure S7). Among these genes, those related to four major oncogenic pathways (ERBB, EGF, JAK-STAT, and Apoptosis) were visualized as heat maps and dot plots (Fig. 6D) [44, 45]. The dot plots displayed the associated GOs for each gene, with GO IDs and descriptions separated by pathway (Fig. 6E). The individual miRNA-treated groups displayed diverse gene expression patterns. However, the 3-miRNA mixed group showed increased patterns in the expression of most genes involved in the four oncogenic pathways. Upon observing significantly increased or decreased mRNA expression patterns in each group compared to the control group through the Disease Ontology (DO) database (Figure S8), lung-related diseases were predominantly observed among all diseases (Fig. 6F). Specifically, upon confirming lung cancer-related DO terms through gene set enrichment analysis (GSEA), significance was confirmed in miR200b_mimic (*p*-value = 0.01), miR92b_inhibitor (*p*-value = 0.001) and the 3-miRNA mixed group (*p*-value < 0.001). Moreover, the mixed group (enrichment score = 0.384) had the highest enrichment score (Fig. 6G). The leading-edge genes associated with lung cancer-related enrichment scores are displayed at each group’s bottom of the GSEA graph. The expression patterns of the related genes are displayed as a heat map (Figure S9). The introduction of the 3-miRNAs was closely associated with lung diseases and may enhance lung tumor initiation and progression.

## Discussion

Although exosomes include various nucleic acids including DNA, mRNA, miRNA and noncoding RNA, our data observed that miRNAs were enriched within exosome. These results were consistent with our previous studies in non-SCLC [17, 18]. Some studies suggested that miRNAs are not randomly selected and loaded into exosome, and exosomes are enriched in unique repertories of miRNAs, rather than a mere reflection of the compositions of their parental cells [46–49]. In addition, the miRNA profiles of exosome were specific to stimuli and cell types, reflecting the status of their parental cells [50, 51]. In based on previous studies, miRNAs within tumor cell-derived exosomes can have specific profiles because of tumor heterogeneity and microenvironment complexity such as hypoxia, acid, and various stimuli [52, 53]. Indeed, our data observed that contents of exosomal miRNA was homogenous in healthy individuals, but was heterogeneous in patients with SCLC. Although tumor-derived exosomes are difficult to specifically isolate from patient blood using the current technology, tumor diagnosis and screening are possible if the characteristics of exosomes, which reflect tumor heterogeneity and specificity, are utilized in liquid biopsy.

Our studies finally selected exosomal miR-200b-3p, miR-3124-5p, and miR-92b-5p for diagnosis of SCLC. The roles of miR-3124-5p and miR-92b-5p have not been revealed yet in oncology field. In addition, miR-200b-3p is one particular member of the miR-200 family, and their roles remained controversial. Some reports demonstrated that miR-200b-3p play their roles as tumor-suppressor gene, especially in inhibiting metastasis. They showed that miR-200b-3p can target against zinc finger E-box-binding homeobox 1/2 (ZEB1/2), microfibril-associated glycoprotein 2 (MAGP2), mothers against decapentaplegic homolog 2 (SMAD2) and high mobility group box 3 (HMGB3), thereby inhibiting tumor growth, apoptosis and cellular mobility [54–57]. However, other reports have pointed out that miR-200b-3p can exert oncogenic functions as showing the promotion of tumor progression by downregulating some tumor suppressor genes, including cassette subfamily A member 1 (ABAC1), large tumor suppressor kinase 2 (LATS2) and transcriptional intermediary factor 1 γ (TIF1γ) [58–60]. In addition, miR-200b-3p may have different functions according to the type of tumor. Although miR-200b exhibits tumor-suppressive effects in a number of tumor types, they mainly acted as an oncogene in lung cancer [58, 60, 61]. In our studies, we did not define the function of each miRNA, and it needs further studies to evaluate the function of each miRNA in SCLC.

Accumulating data has demonstrated the application of miRNAs as prognostic and diagnostic factors for various diseases, particularly through circulating or exosomal miRNAs. While many studies have suggested using single cellular miRNA, recent reports have highlighted miRNA panels consisting of combinations of miRNAs as promising biomarkers for prognosis and diagnosis. Our data showed that the specificity of SCLC diagnosis was further increased with miRNA combinations compared to individual miRNAs alone. Furthermore, our miRNA panel was found to be associated with various oncogene pathways and nervous system development, as revealed by KEGG and GO analysis. Given that SCLC is of neuroectodermal origin and expresses neuroendocrine differentiation markers, our miRNA panel may be associated with the progression and tumorigenesis of SCLC. We believe that our miRNA panel provides new insights into the mechanisms of SCLC pathogenesis.

In summary, our investigation has identified SCLC-specific exosomal miRNAs, which have been evaluated as a possible prognostic marker for SCLC. Among these miRNAs, the 3-miRNA panel (miR-200b-3p, miR-3124-5p, and miR-92b-5p) may serve as a diagnostic and prognostic marker for SCLC. To improve the diagnostic accuracy of the 3-miRNA panel, further validation in a larger cohort and a better understanding of the pathophysiologic functions of these miRNAs are required.

### Electronic supplementary material

Below is the link to the electronic supplementary material.

Supplementary Material 1

## Data Availability

RNA sequencing data for exosomal miRNA-seq (GSE232903) derived from the sera of SCLC patients generated in this study and mRNA-seq (GSE232904) derived from the BEAS2B cell line transduced with miRNAs were deposited in the NCBI Gene Expression Omnibus (http://www.ncbi.nlm.nih.gov/geo). The remaining data are available within the article and supplementary material.

## Abbreviations

NSCLC: Non-small cell lung cancer
SCLC: Small cell lung cancer
ED: Extensive disease
LD: Limited disease
miRNA: MicroRNA
AUC: Area under the curve
ROC: Receiver operating characteristics
