# Supplementary material for: Identification of exosomal microRNA panel as diagnostic and prognostic biomarker for small cell lung cancer
Source: Biomark Res. 2023 Sep 13;11:80. doi: 10.1186/s40364-023-00517-1 (PMC10500735; doi:10.1186/s40364-023-00517-1)
Supplement: Supplementary file 1 — Supplementary Material 1 [file 40364_2023_517_MOESM1_ESM.docx]

**Supplementary Materials**

**Identification of exosomal microRNA panel as diagnostic and prognostic biomarker for**

**small cell lung cancer**

Dong Ha Kim^1^, Hyojeong Park^2^, Yun Jung Choi^1^, Kyungtaek Im^1^, Chae Won Lee^2^, Da-Som Kim^2^, Chan-Gi Pack^3^, Hyun-Yi Kim^4^, Chang-Min Choi^5,6^, Jae Cheol Lee^6^, Wonjun Ji^5,*^, Jin Kyung Rho^3,*^

^1^Asan Institute for Life Sciences, ^2^Department of Biomedical Sciences, AMIST, ^3^Department of Convergence Medicine, ^5^Department of Pulmonology and Critical Care Medicine, and ^6^Department of Oncology, Asan Medical Center, University of Ulsan College of Medicine, Seoul 05505, South Korea

^4^NGeneS Inc., Asan-Si, Gyeonggi-do, South Korea

*Co-corresponding authors:

Wonjun Ji

Department of Pulmonary Critical and Care Medicine, University of Ulsan College of Medicine, 88, Olympic-ro 43-gil, Songpa-gu, Seoul 05505, South Korea. Tel.: +82 2 3010 1699; Fax: +82 2 3010 6961; E-mail: jack1097@naver.com

Jin Kyung Rho

Department of Convergence Medicine, University of Ulsan College of Medicine, 88, Olympic-ro 43-gil, Songpa-gu, Seoul 05505, South Korea. Tel.: +82 2 3010 2974; Fax: +82 2 3010 6961; E-mail: jkrho@amc.seoul.kr

**Supplementary figures**


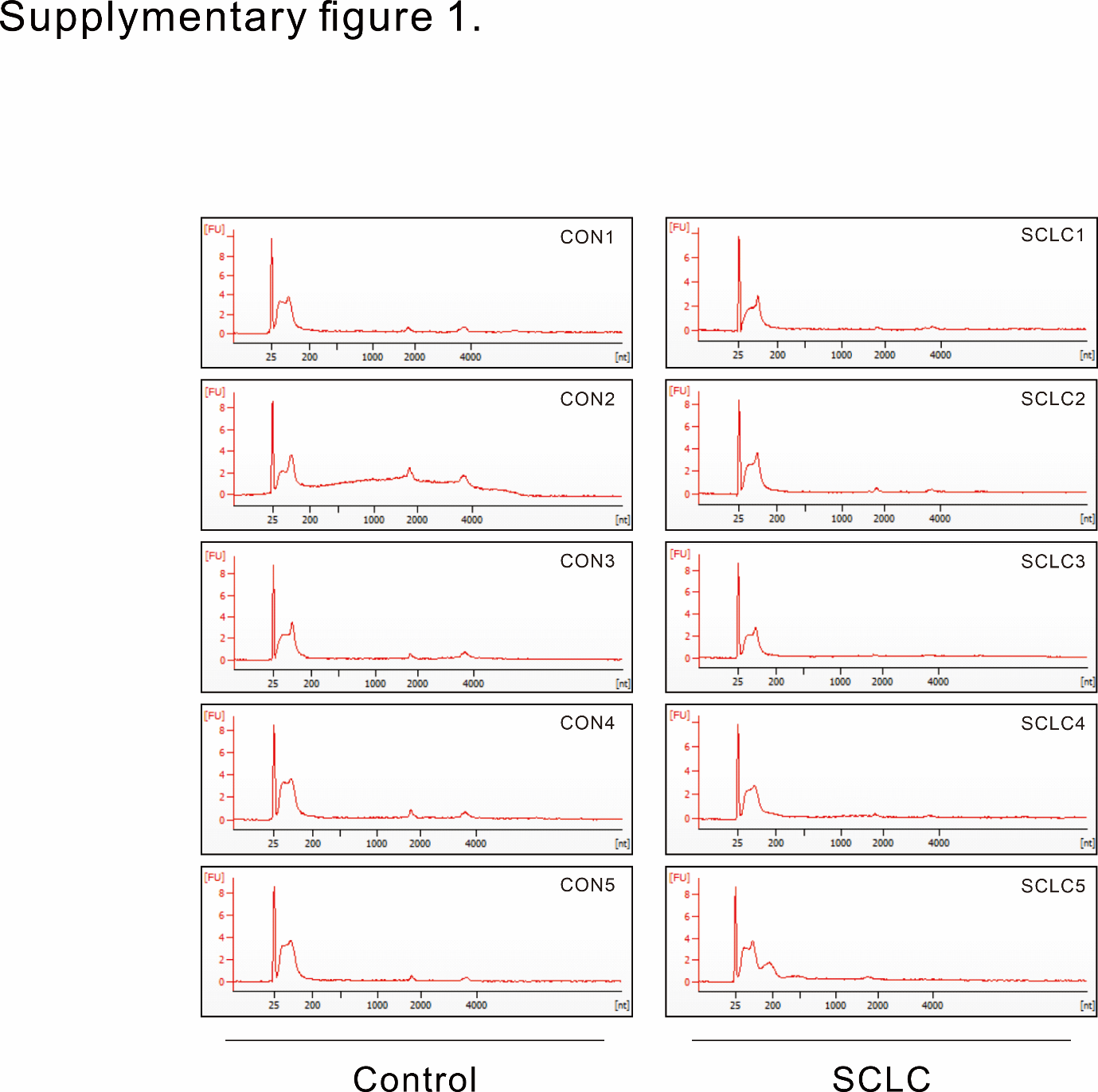


**Supplementary Figure 1.** Bioanalyzer electropherograms profiles of exosomal total RNA from non-cancerous lung nodule and patients with small cell lung cancer (SCLC).

**
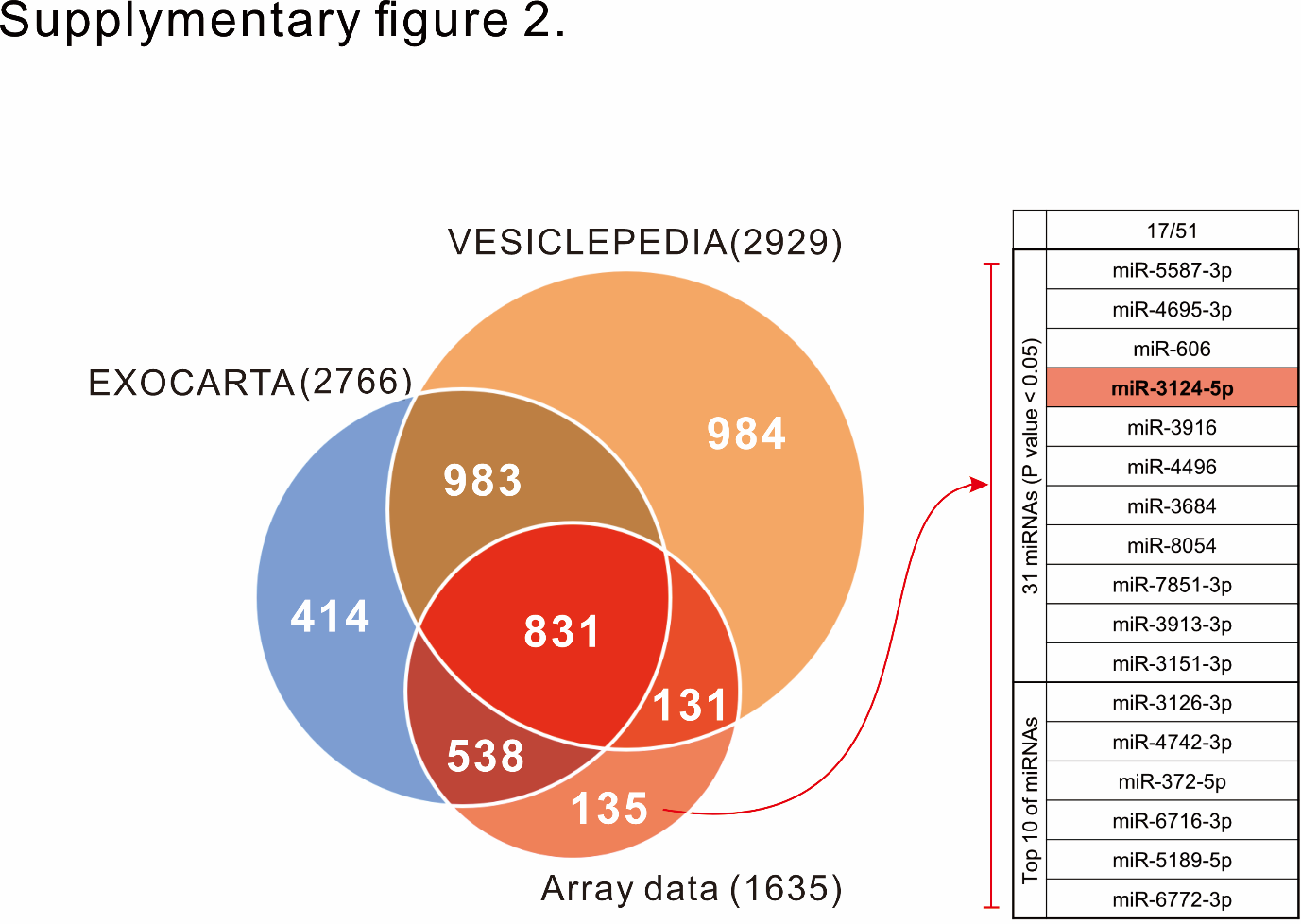
**

**Supplementary Figure 2.** Venn diagrams show the number of all miRNAs differentially expressed between Vesiclepedia and ExoCarta exosomal miRNA databases to compare our miRNA array data sets.

**
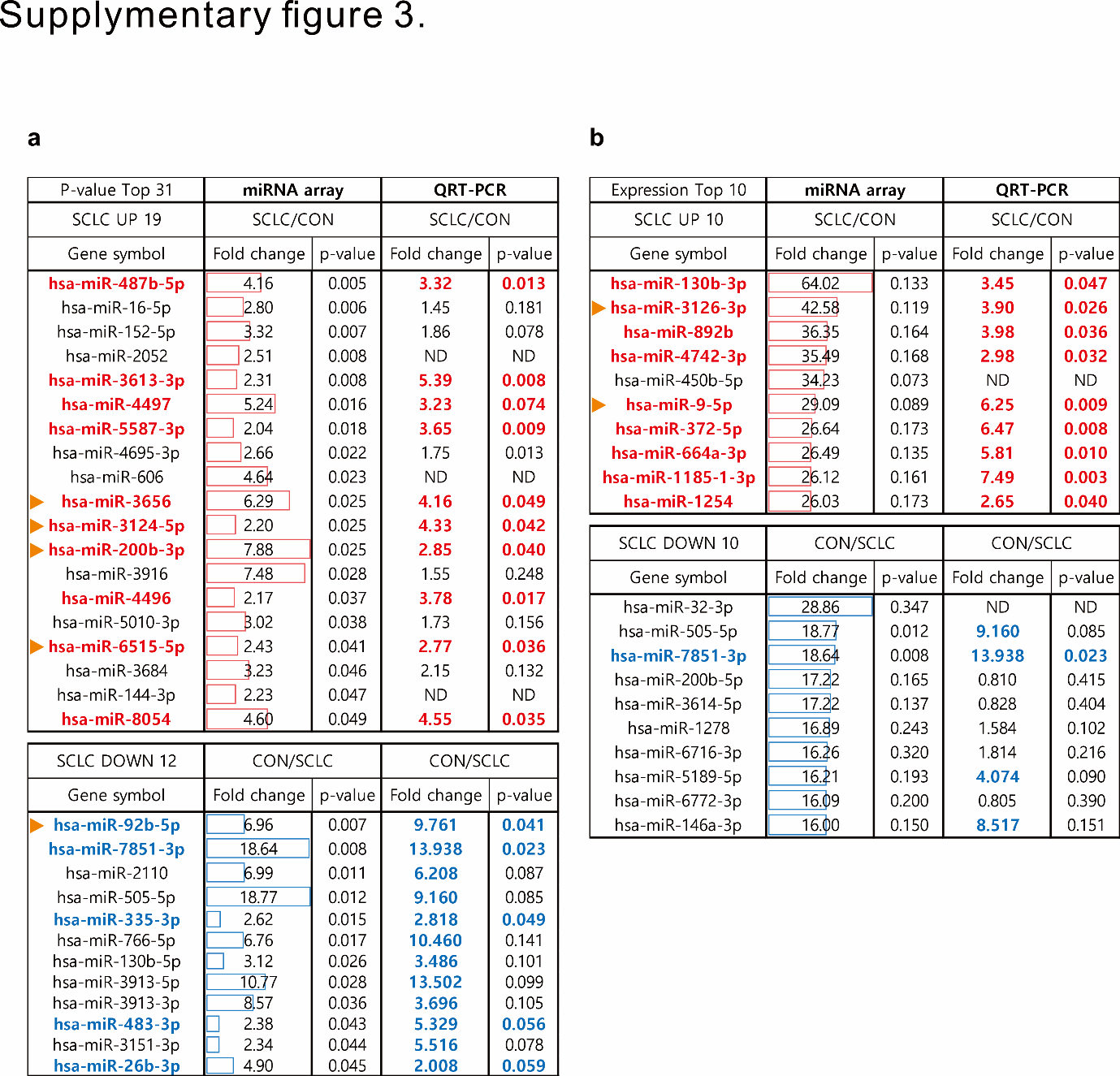
**

**Supplementary Figure 3.** Numerical results of miRNA array data and qRT-PCR results. (**a**) Significantly more than 2-fold up- and down-regulated 31 exosomal miRNAs (*p* < 0.05). (**b**) Ten exosomal miRNAs; each with the highest or lowest expression rates in patients with SCLC compared to non-cancerous lung nodules (controls). The 25 miRNAs validated in the "Analytic set" are shown in red and blue (red: upregulated, blue: downregulated), and the seven miRNAs validated in the "Test set" are indicated by orange arrows.

**
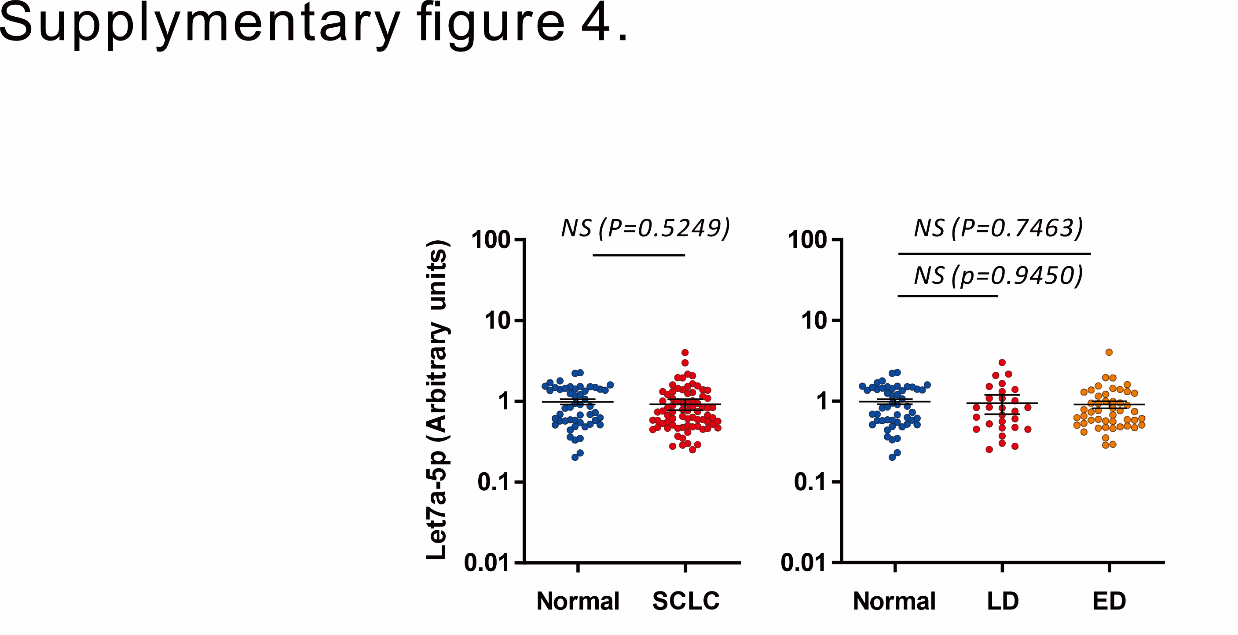
**

**Supplementary Figure 4.** Validation of the reference gene within the exosomes. Quantitative real-time PCR was performed using serum-derived exosomes from 28 limited disease (LD)-small cell lung cancer (SCLC), 48 extensive disease (ED)-SCLC, and 50 healthy participants. The expression level of exosomal internal control Let-7a-5p was assessed via real-time PCR analysis.

**
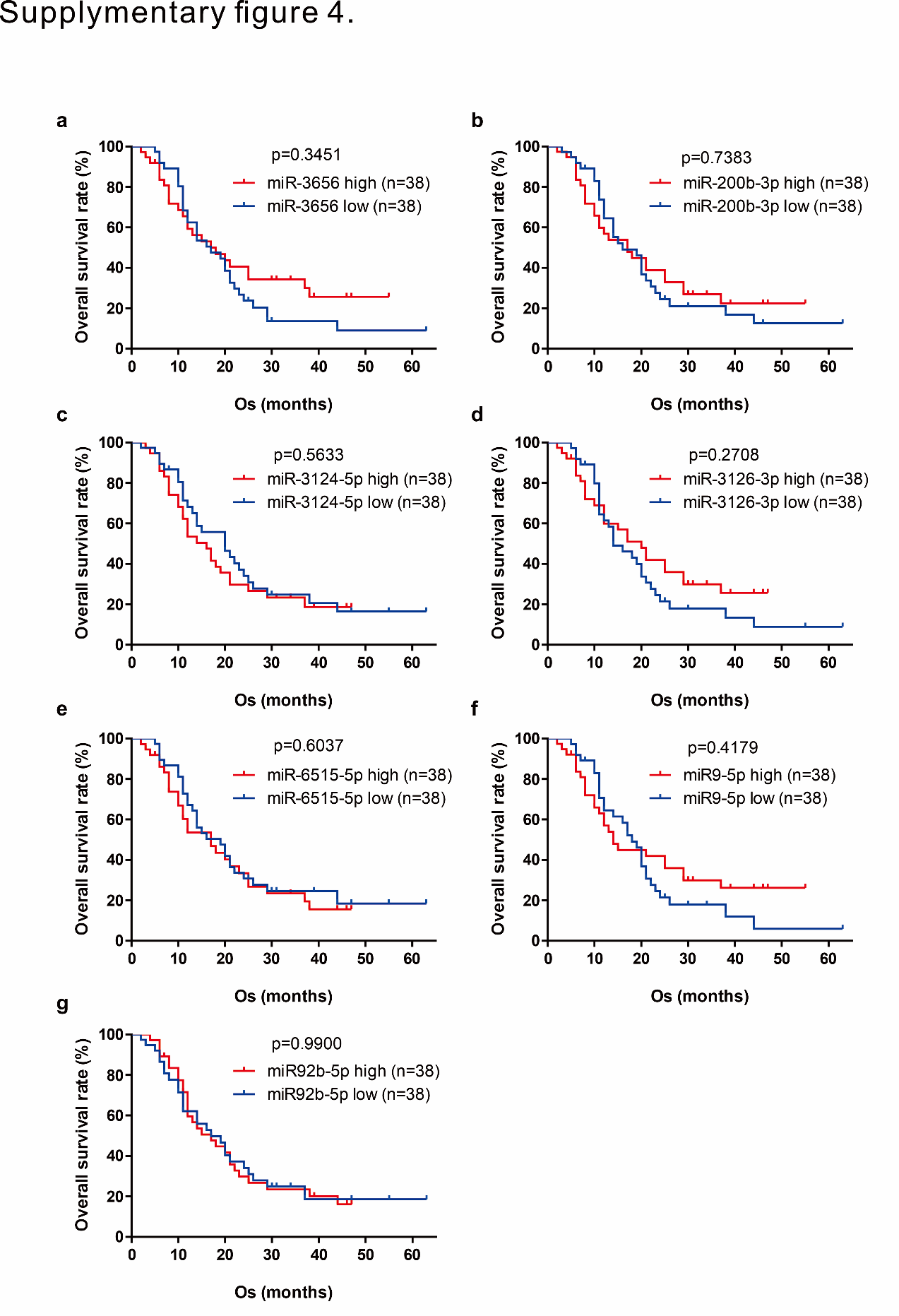
**

**Supplementary Figure 5.** Kaplan–Meier survival curve stratified by high and low (**a**) miR-3656 (**b**) miR-200b-3p; (**c**) miR-3124-5p; (**d**) miR-3126-3p; (**e**) miR-6515-5p; (**f**) miR-9-5p, and (**g**) miR-92b-5p separately exosomal miRNAs expression level.

**
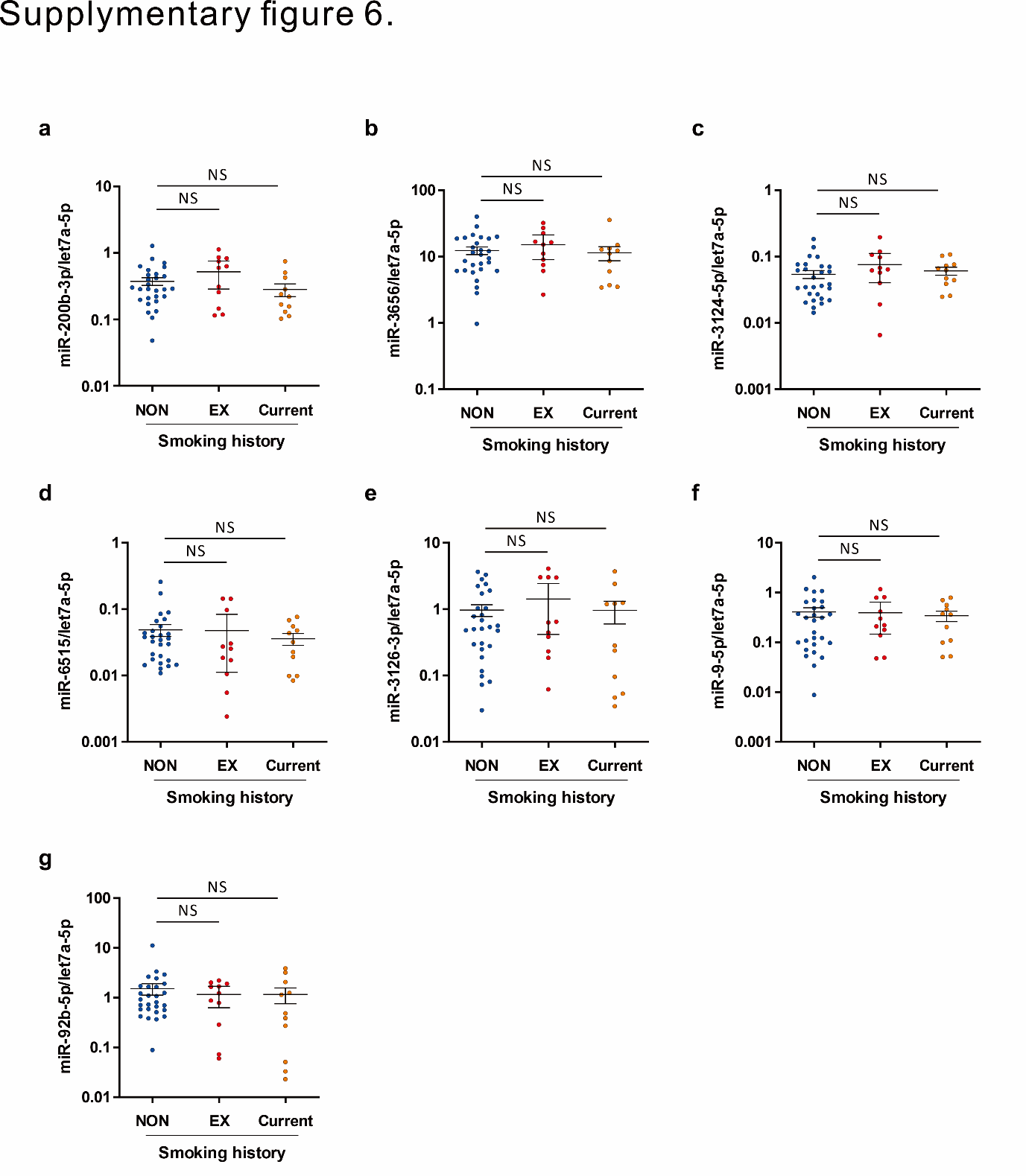
**

**Supplementary Figure 6.** Quantitative real-time PCR was performed using serum-derived exosomes from 50 healthy participants, including 28 non-smokers (NON), 11 ex-smokers (EX; no longer smoking), and 11 current smokers (Current). (**a**) Expression levels of hsa-miR-200b-3p; (**b**) hsa-miR-3656; (**c**) hsa-miR-3124-5p; (**d**) hsa-miR-6515; (**e**) hsa-miR-3126-3p; (**f**) hsa-miR-9-5p, and (**g**) hsa-miR92b-5p. Let-7a-5p was used as an internal control.

**
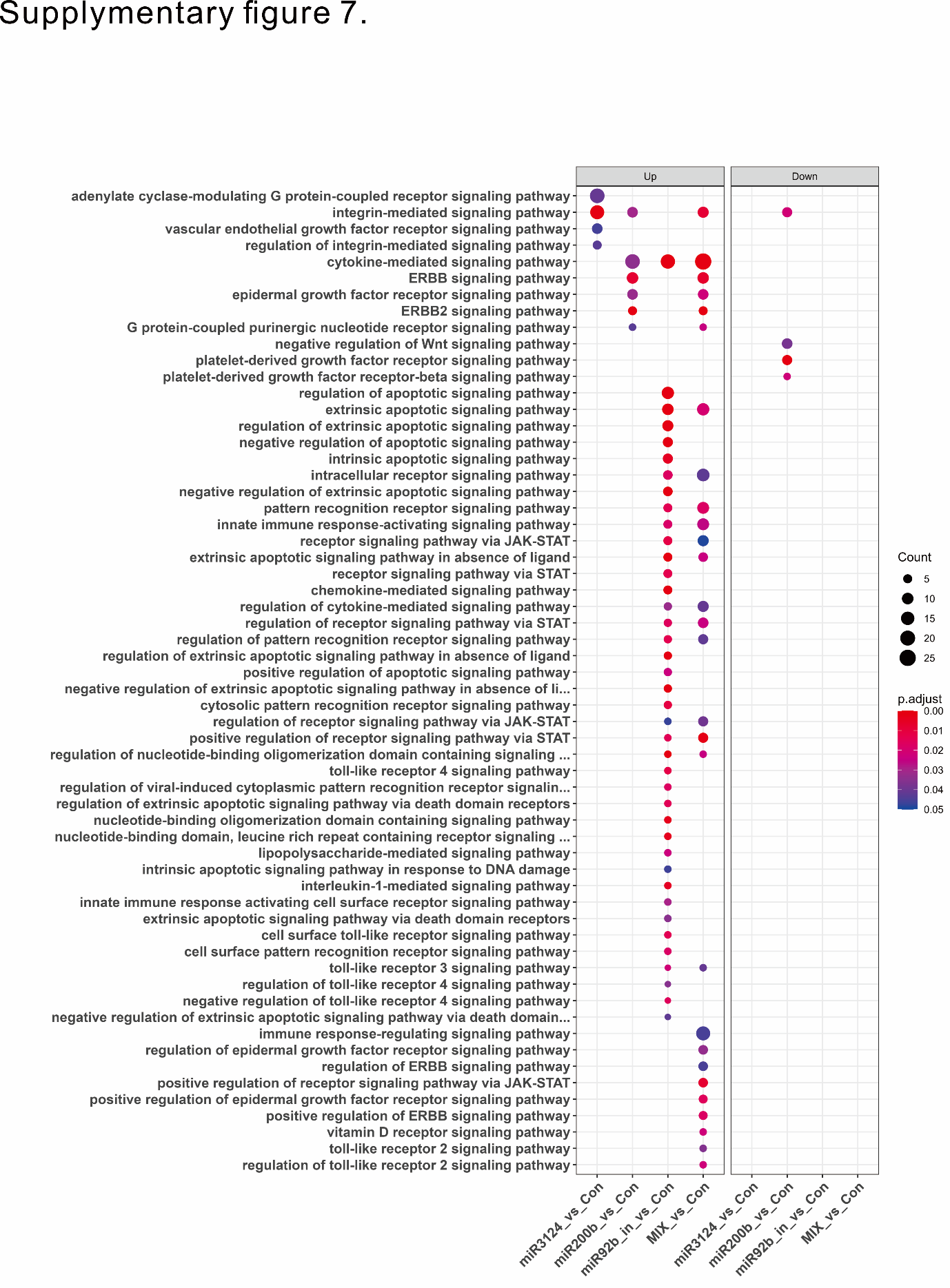
**

**Supplementary Figure 7.** Pathway related gene ontology (GO) analysis of significantly increased or decreased mRNAs by the miRNA treatment group (fold change cut-off of 2 and *p*-value cut-off of 0.05).

**
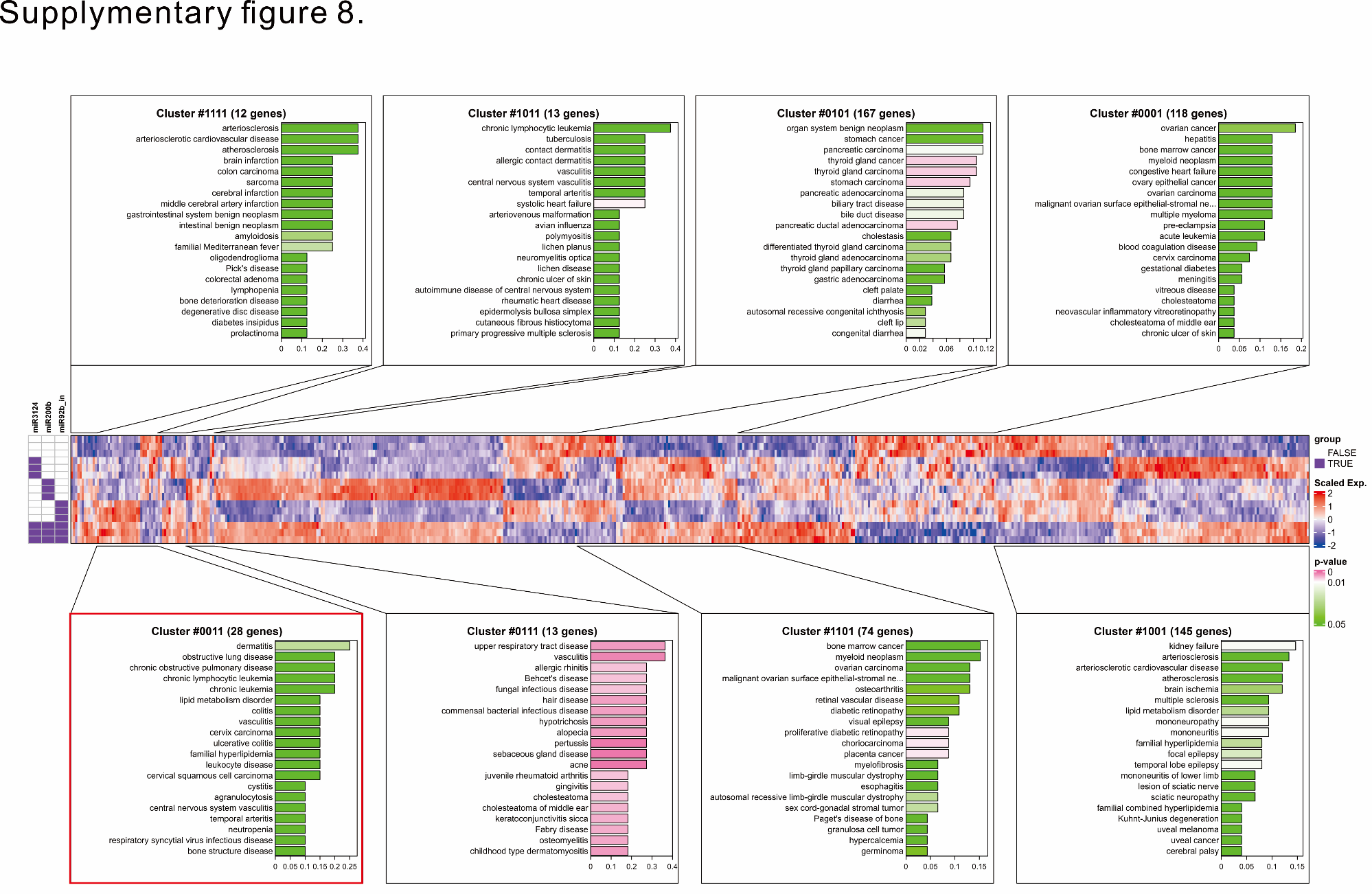
**

**Supplementary Figure 8.** Heatmap and DO (Disease Ontology) cluster of significantly increased or decreased mRNAs by the miRNA treatment group (fold change cut-off of 2 and *p*-value cut-off of 0.05).

**
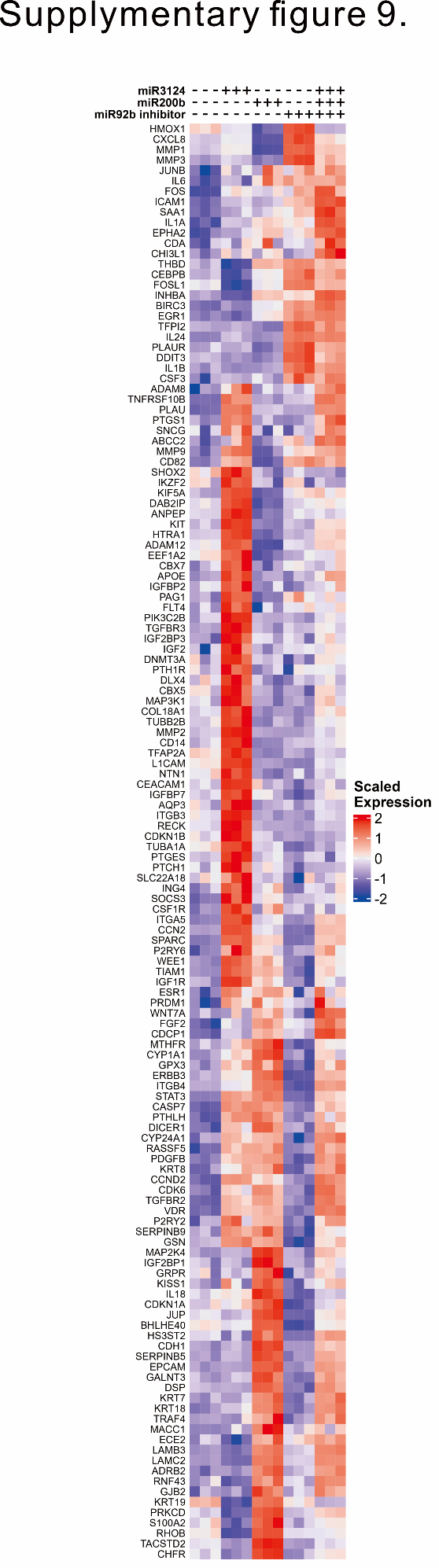
**

**Supplementary Figure 9.** Heatmap analysis of leading-edge genes from the lung cancer-related disease ontology (DO) terms.

**Supplementary Table 1.** Primer sequences.
